# Groundwater and soil moisture data collected in the coastal critical zone of the Delmarva Peninsula, United States

**DOI:** 10.1016/j.dib.2025.111336

**Published:** 2025-01-28

**Authors:** Dannielle Pratt, Rachel W. McQuiggan, Eva Snell Bacmeister, Amanda Sprague-Getsy, Holly A. Michael

**Affiliations:** a Department of Civil, Construction and Environmental Engineering, Pierre S. Dupont Hall, 127 The green, Newark DE 19716, United States; b Delaware Geological Survey, 257 Academy Street, Newark, DE 19716, United States; c Department of Earth Sciences, 255 Academy Street, Newark, DE 19716, United States

**Keywords:** Hydrology, Salt marsh, Saltwater intrusion, Sea level rise, Time series

## Abstract

Data presented in this paper were collected from three agricultural and three forested sites along the Delmarva Peninsula, United States between March 2021 and July 2024. The six study sites border salt marsh and have experienced agricultural loss or forest loss from marsh migration driven by sea-level rise, saltwater and freshwater flooding events, and droughts. The study sites were subdivided into 4 zones each: healthy, marginal, transitional, and marsh. Hydrological monitoring equipment was installed in each zone to study space-for-time changes from healthy marsh to healthy upland. Groundwater level, conductivity, and temperature; and soil moisture, conductivity, and temperature were measured in each zone in 15-minute intervals to understand changes in the shallow aquifer and root zone soils. These high-resolution measurements capture critical processes in the shallow aquifer and root-zone soils, including shallow soil salinization during storm surges, groundwater salinization during droughts, and aquifer flushing during rainfall events. This data is crucial for understanding the drivers of marsh migration and their associated feedback mechanisms in the coastal critical zone.

Specifications Table

**Table:** 

Subject	Environmental Science
Specific subject area	Hydrology
Type of data	Time Series: Raw, Analysed, Filtered, Processed
Data collection	Groundwater: Groundwater pressure, temperature, and conductivity data were collected with Solinst M3001 conductivity, temperature and depth (CTD) Leveloggers and barometric pressure collected with Solinst Barologgers. Groundwater pressure was converted to depth-to-water using the barometric pressure and manual beep tape measurements. Electrical conductivity was converted to specific conductance using the temperature data from the loggers. Soil Moisture: Soil water content, saturated extract, and temperature data were collected with METER Group TEROS 12 sensors and ZL6 dataloggers.
Data source location	Institution: University of DelawareCity/Town/Region: 1. Milford (DE) 2. Dover (DE) 3. Princess Anne (MD) 4. Crisfield (MD) 5. Nassawadox (VA) 6. Metompkin (VA)
Data accessibility	Repository Name: Hydroshare Direct URL to data: https://www.hydroshare.org/resource/845670d788eb4385a17b1699bdc0383d/

## 1 Value of the Data

• These datasets characterize groundwater level, temperature, and specific conductance; soil water content, temperature, and saturation extract in three coastal agricultural and three coastal forest sites along the Delmarva Peninsula. Sea-level rise, storm surges, and other macroclimatic drivers (e.g., droughts and increased frequency of spring rain events), are driving coastal marshes to migrate upland into healthy forests and agricultural lands.
• Coastal marsh systems are under-studied because data collection is often hindered by high salinity affecting sensors, storms, flooding, and other coastal hazards destroying equipment, and the remote nature of the sites.
• The data presented in this publication are high-resolution timeseries datasets in 15-min intervals that capture the short- and long-term impacts of hydrology driven changes to shallow groundwater and unsaturated zone soils. This data collection was designed along transects from healthy marsh to healthy upland to capture space-for-time processes of marsh migration.
• The data provide a detailed view of the changes occurring as uplands die off and marshes move inland. These datasets capture the hydrology within healthy marshes, transitioning uplands, and healthy uplands, which is crucial to understanding the spatial and temporal nature of landscape changes.
• The data in this study is valuable not only for analysis of present-day regime shifts along the Delmarva, but also to inform hydrological models to predict future shifts in coastal settings globally.

## 2 Background

**Fig. 1:**
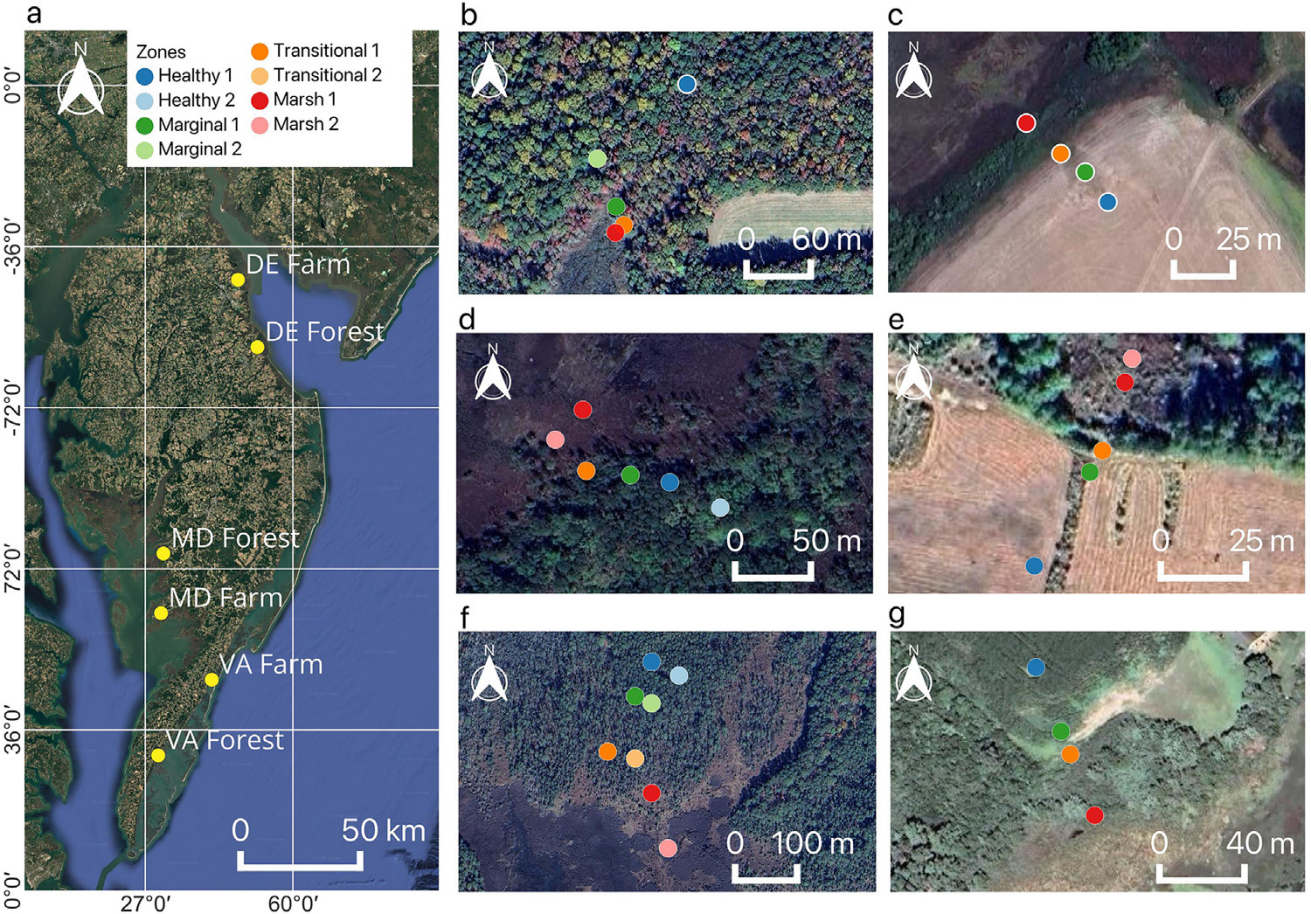
Map of the study sites and zones a) Six coastal CZN sites along the Delmarva, b) DE Forest, c) DE Farm, d) MD Forest, e) MD Farm, f) VA Forest, g) VA Farm. Colors of the zones on the map correspond to the colors in the figures below.

The Coastal Critical Zone network chose study sites with differing land uses, topographic slopes, and coastal exposures to capture varying responses to hydrological drivers of marsh migration. One forest and one farm site bordering salt marsh were chosen in each of Delaware, Maryland, and Virginia (Fig. 1a). The sites were instrumented in transects spanning from healthy saltmarsh to healthy upland and delineated into zones of varying vegetation health: a) healthy: vegetation is alive and there are no visible signs of salt stress; b) marginal: some forest trees are visibly stressed (loss of leaves) and crops in the agricultural fields are smaller and patchy; c) transitional: many of the adult trees are dead while shrubs and saplings dominate the understory; there are open patches of field where crops are no longer grown, and salt crusts may be visible on the soil surface; d) marsh: dominated by *Spartina patens* and/or *Phragmites australis* and fine silt and mud sediments (Fig. 1b–g).

Average tidal range at the Delaware and Maryland sites is ∼0.4 m and at the Virginia sites is ∼ 2 m relative to sea level (NAVD88) [[1], [2], [3]] and the sites experience frequent storms due to the humid subtropical climate in the Mid-Atlantic. Most major storms occur in the fall and winter months (Sep-Jan) and droughts are most prevalent in the summer and early fall (Jun-Sep). As shown in this dataset, storm surges and overland saltwater flooding from storms, most notably Tropical Storm Wanda (October 2021), drive increases in shallow root zone salinity and moisture content that can take up to 6 months to recover, depending on the soil type. Additionally, droughts can drive significant shallow subsurface salinization (Summer 2022) due to the reversal of flow in the groundwater.

The forest sites are dominated by *pinus taeda* (Loblolly Pines) and agricultural fields are dominated by either soybean or corn for half of the year on biennial rotation. The pines, corn, and soybean have a low conductivity tolerance, between 1 and 3 mS/cm [4,5] and vegetation die-off can be observed at the sites.

## 3 Data Description

**Fig. 2:**
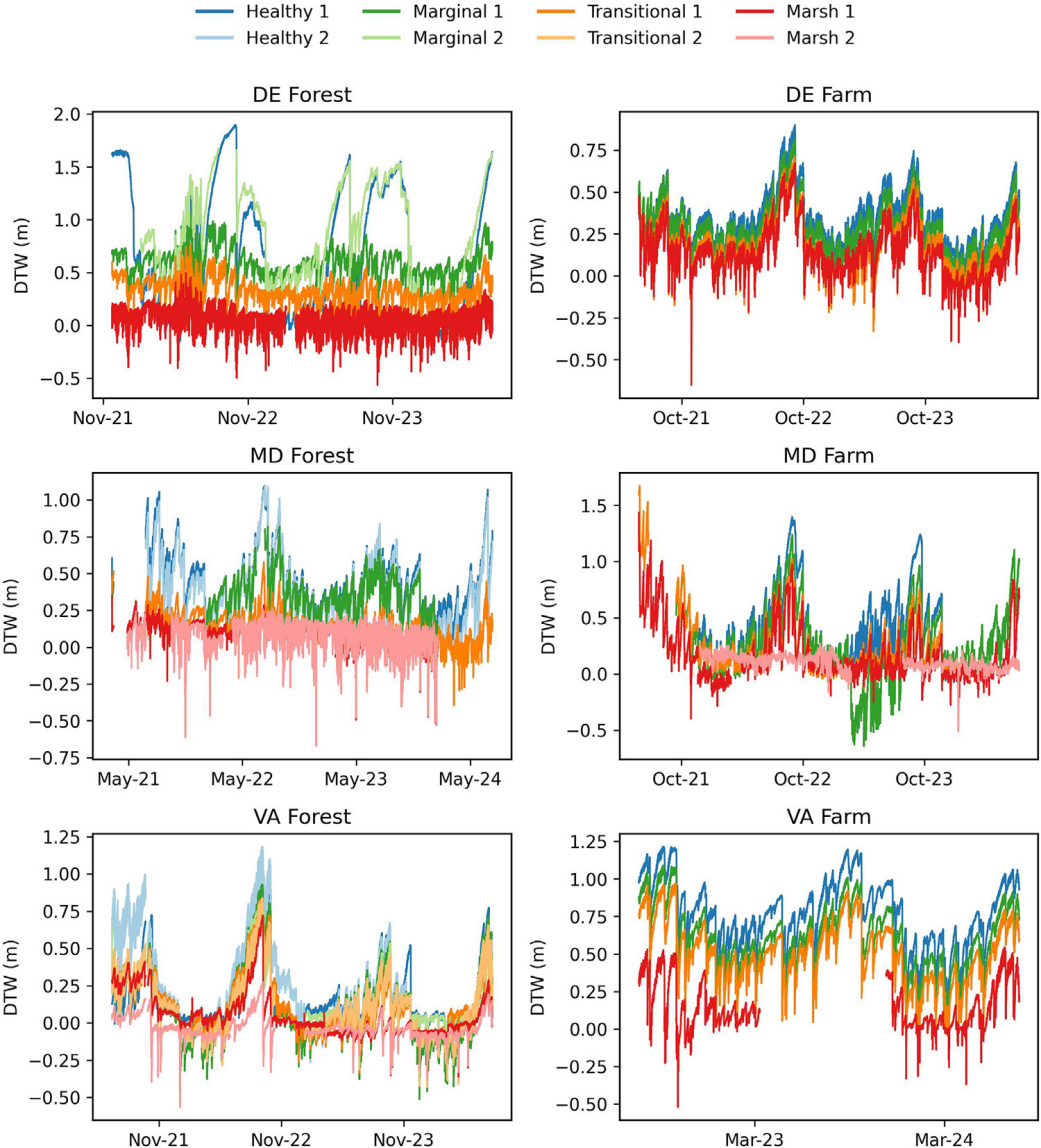
Groundwater data (depth to water) for DE Forest (November 2021 to July 2024), DE Farm (May 2021 to July 2024), MD Forest (March 2021 to July 2024), MD Farm (May 2021 to July 2024), VA Forest (June 2021 to July2024), and VA Farm (July 2022 to July 2024).

**Fig. 3:**
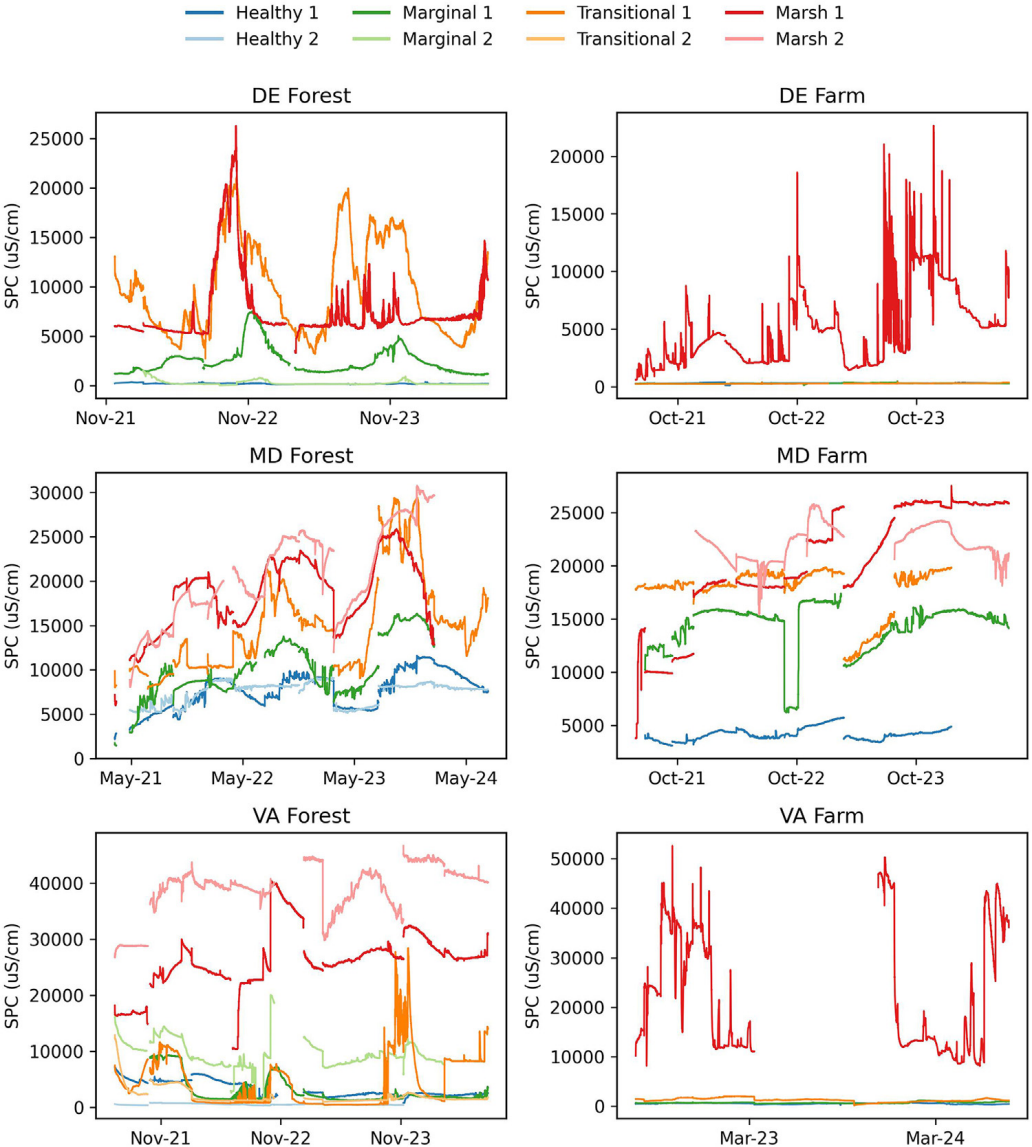
Groundwater specific conductance for DE Forest (November 2021 to July 2024), DE Farm (May 2021 to July 2024), MD Forest (March 2021 to July 2024), MD Farm (May 2021 to July 2024), VA Forest (June 2021 to July2024), and VA Farm (July 2022 to July 2024).

**Fig. 4:**
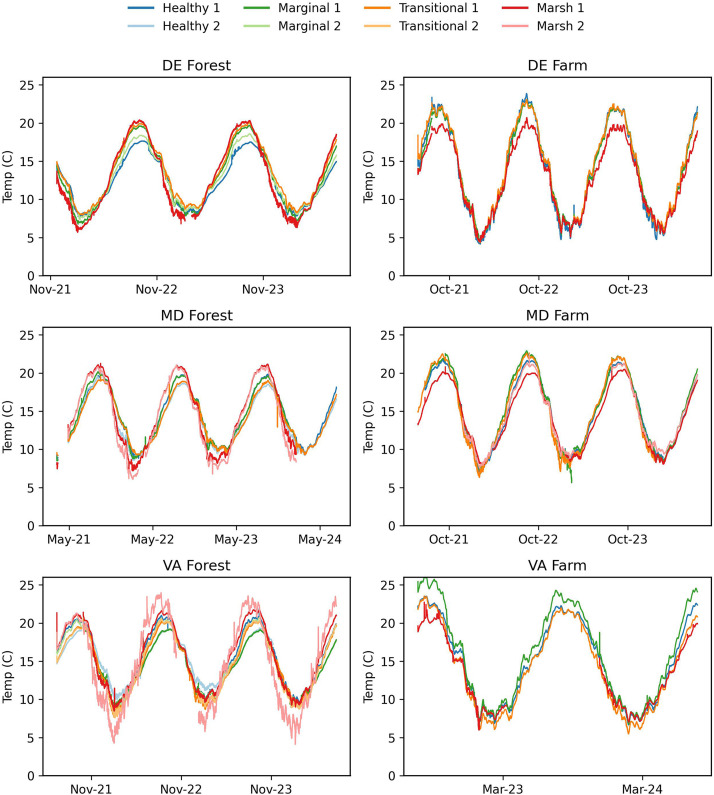
Groundwater temperature for DE Forest (November 2021 to July 2024), DE Farm (May 2021 to July 2024), MD Forest (March 2021 to July 2024), MD Farm (May 2021 to July 2024), VA Forest (June 2021 to July2024), and VA Farm (July 2022 to July 2024).

Fig. 2, Fig. 3, Fig. 4 show the groundwater level (depth to water, m), specific conductance (μS/cm), and temperature (degrees C) collected from the shallow wells with Solinst conductivity, temperature, and depth (CTD) sensors. The depths of the wells and ground surface elevation are provided in the metadata on Hydroshare for each site. Temperature data have not undergone any user processing and are direct output as downloaded from the Solinst Levelogger software. Pressure data were corrected for barometric fluctuations using the Solinst Levelogger software, then converted to depth-to-water using reference depth to water measurements made at the start and stop of each deployment using a Solinst beep tape. Electrical conductivity measurements were converted to specific conductance using the standard equation given by Solinst (2024a, 2024b) [6,7]. Collection dates of the groundwater data for each site are as follows: 1) DE Forest: November 2021-July 2024, 2) DE Farm: May 2021-July 2024, 3) MD Forest: May 2021-July 2024, 4) MD Farm: May 2021-July 2024, 5) VA Forest: June 2021-July 2024, 6) VA Farm: July 2022-July 2024. Groundwater data are reported in the Hydroshare repository within its designated site's resource as “SiteName_DTW, Temperature, Specific conductance- Start Date-EndDate”. Within the data resource, there is an XXSites.csv file which describes the site naming system, well locations, installation dates, well screen depths, and zone type. There is also a “SiteName_DTW_SC_TE_StartDate_EndDate.csv” file where the sensor data are located. Additionally, a ReadMe.md file is provided with additional study method, site, and data descriptors. Data in Hydroshare is available from Pratt et al. (2025) [8].

**Fig. 5:**
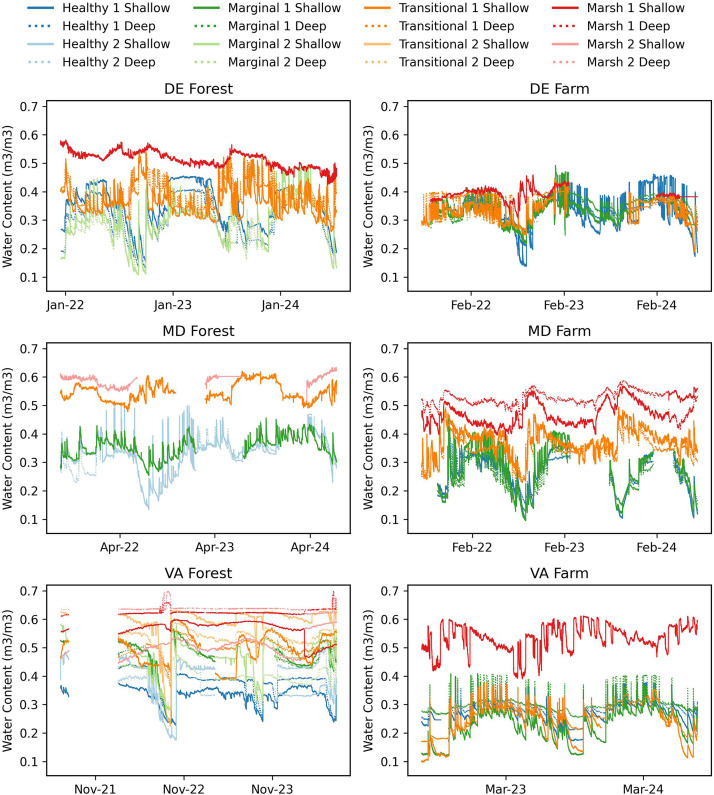
Soil Moisture from for DE Forest (December 2021 to July 2024), DE Farm (July 2021 to July 2024), MD Forest (August 2021 to July 2024), MD Farm (July 2021 to July 2024), VA Forest (June 2021 to July 2024), and VA Farm (July 2022 to July 2024).

**Fig. 6:**
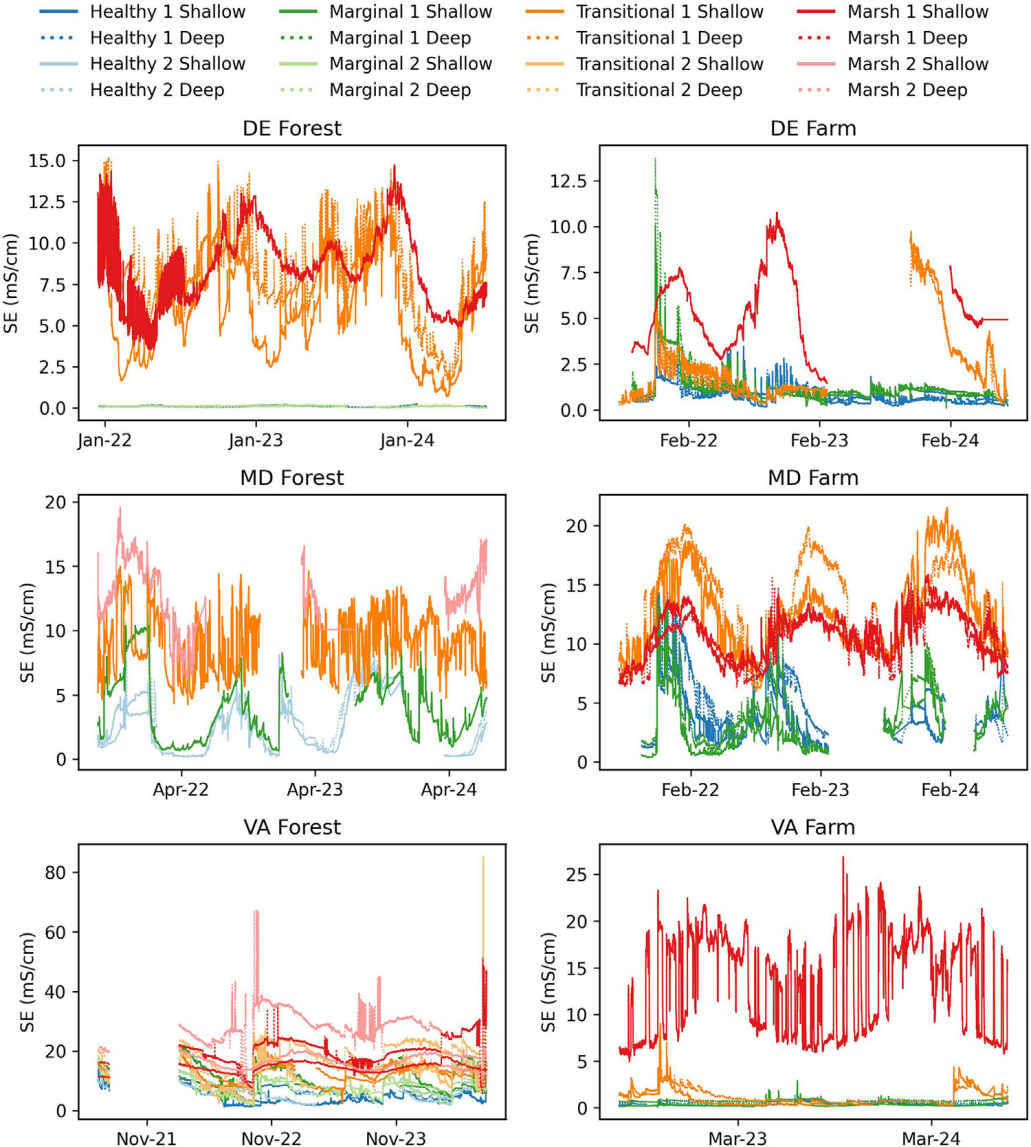
Saturated extract for DE Forest (December 2021 to July 2024), DE Farm (July 2021 to July 2024), MD Forest (August 2021 to July 2024), MD Farm (July 2021 to July 2024), VA Forest (June 2021 to July 2024), and VA Farm (July 2022 to July 2024).

**Fig. 7:**
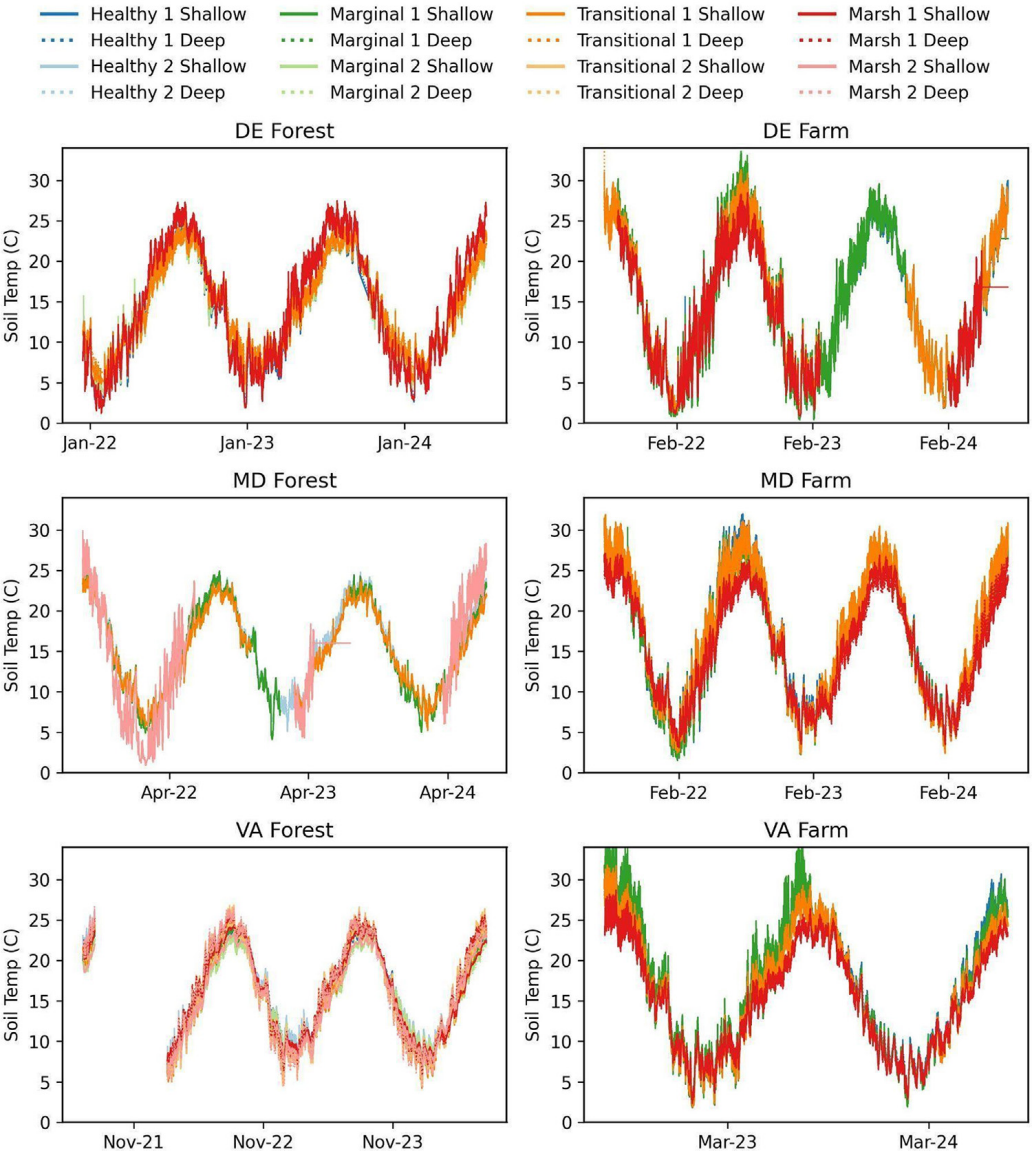
Soil temperature for DE Forest (December 2021 to July 2024), DE Farm (July 2021 to July 2024), MD Forest (August 2021 to July 2024), MD Farm (July 2021 to July 2024), VA Forest (June 2021 to July 2024), and VA Farm (July 2022 to July 2024).

Fig. 5, Fig. 6, Fig. 7 show the soil moisture water content (m^3^/m^3^), saturation extract (mS/cm), and temperature (degrees C) collected from the shallow and deep Teros 12 soil sensors and ZL6 dataloggers. Shallow sensors were installed from 10 to 20 cm below the surface and deep sensors installed 20–30 cm below the surface. Collection dates of the soil data for each site are as follows: 1) DE Forest: December 2021-July 2024, 2) DE Farm: July 2021-July 2024, 3) MD Forest: August 2021-July 2024, 4) MD Farm: July 2021-July 2024, 5) VA Forest: June 2021-July 2024, 6) VA Farm: July 2022-July 2024. Soil data is reported in the Hydroshare repository within its designated site's resource as “SiteName_Soil water content, Soil temperature, Electrical conductivity- Start Date-EndDate”. Within the data resource, there is an "XXSites.csv" file which describes the site naming system, sensor locations, installation dates, sensor depths, and zone type. There is also a “SiteName_WC_ST_SC_StartDate_EndDate.csv” file where the sensor data is located. Additionally, a ReadMe.md file is provided with additional study method, site, and data descriptors. Data in Hydroshare is available from (Pratt et al., 2025) [8].

## 4 Experimental Design, Materials and Methods

### 4.1 Field design

#### 4.1.1 Groundwater

Each zone was outfitted with 1.5-in. (3.8 cm) diameter monitoring piezometers constructed of PVC with 12-in. (30.5 cm) screen intervals. Conductivity, temperature, and depth (CTDs) loggers (Solinst M3001 Levelogger 5) were installed in each well to monitor groundwater at 15-min intervals. Specific conductance (SPC) was considered a proxy for salinity. A barometric logger (Solinst M3001 Barologger 5) was installed at every site to compensate the groundwater levels for atmospheric pressure [9,10].

#### 4.1.2 Soil moisture

Each zone was outfitted with a TEROS 12 3-in-1 soil moisture + temperature & EC sensor from METER Group. Healthy, marginal, and transitional zones at every site were outfitted with a shallow sensor (10–20 cm depth) and a deep sensor (20–30 cm depth), while most marsh zones were only outfitted with a shallow sensor. The Maryland agriculture and Virginia forest sites have both a shallow and deep sensor in their marsh zones. Cores were augered to 30 cm, sensors inserted, and the hole backfilled with the same sediment to not change the soil type. Water content, saturation extract, and soil temperature was collected with a ZL6 datalogger from METER Group in 15-minute intervals.

### 4.2 Data collection, post-processing, and cleaning

Depth-to-water was measured according to the Delaware Geological Survey groundwater monitoring procedures [10]. Depth-to-water measurements taken manually every time data was downloaded using the Solinst Model 101 P2 Water Level Meter and incorporated into the data processing code to calculate the error of the water level measurements from the conductivity, temperature, and depth sensors (CTDs) due to sensor drift. The CTDs were also removed and calibrated with every data download. Real time conductivity measurements were recorded with 5000 and 12,800 μS/cm calibration solutions in the Solinst software before calibration to quantify sensor drift. The real time values were used to adjust the conductivity values collected from the CTDs to account for drift. The conductivity data was also adjusted for temperature changes and converted to specific conductance in post processing [6,7]. Electrical conductivity measurements were converted to specific conductance using the standard equation given by Standard Methods 2510B [7]. CTDs measured absolute pressure of the groundwater above the sensor (water pressure + barometric pressure) and the total head of the water was calculated by barometric pressure adjustments and ground surface elevation measurements [9,10]. The real-time values from beep tape measurements before deployment and after collection were used to adjust the depth to water values collected from the CTDs to account for drift. Data validation and quality assurance: All depth-to-water values for each logger deployment were assigned an accuracy value. Accuracy values are the difference, in meters, between the last logger-measured depth-to-water value in each deployment and the manual depth to water measurement made at that time. An accuracy value of 0.075 m was used as a threshold and data from deployments with accuracy values of greater than 0.075 m were not included in this dataset.

The soil moisture sensors are not sensitive to variations in soil texture because they run at high measurement frequency [11]. Therefore, its generic calibration equation was used for both volumetric water content (VWC) and bulk electrical conductivity (EC) measurements. The accuracy for VWC measurements is ± 0.03 m^3^/m^3^ and for EC (± 5 % + 0.01 mS/cm) from 0 to 10 mS/cm and ± 8 % from 10 to 20 mS/cm [12]. Most of our data falls below the 20 mS/cm threshold for error, except for the VA Farm and VA Forest where some zones exceed this value. It should be noted that there may be a larger error in any zones where the saturated extract is above 20 mS/cm. The sensors convert to saturated extract from the measured EC.

## Limitations

Missing data values in all the datasets are either due to malfunction of the sensors, battery issues and lost data, time between data deployments when the sensor was removed for calibration or data retrieval, or data that did not meet our data quality control and validation requirements and was removed.

## Ethics Statement

The authors have read and follow the ethical requirements for publication in Data in Brief and confirm that the current work does not involve human subjects, animal experiments, or any data collected from social media platforms.

## CRediT Author Statement

**Dannielle Pratt:** Writing, Investigation, Visualization, Conceptualization Formal analysis; **Rachel McQuiggan:** Methodology, Data curation, Writing - Original Draft, Writing- Review & Editing; **Eva Snell Bacmeister**: Investigation, Writing- Review & Editing; **Amanda Sprague-Getsy**: Investigation, Writing- Review & Editing; **Holly A. Michael**: Conceptualization, Resources, Writing- Review & Editing, Supervision, Funding acquisition

## Data Availability

CUAHSI HydroshareCoastal Critical Zone Dataset: Groundwater and soil moisture data collected in the Coastal Critical Zone of the Delmarva Peninsula, United States (Original data).

## References

[1] U.S. Geological Survey. (2012). *Gage height dataset: chincoteague Bay Inlet at Chincoteague, VA-01484716* . USGS current water data for the nation. https://waterdata.usgs.gov/monitoring-location/01484746/#parameterCode=00035&period=P7D&showMedian=true.

[2] U.S. Geological Survey. (2018). *Gage height dataset: little Annemessex River at Crisfield, MD-01485755* . USGS current water data for the nation. https://waterdata.usgs.gov/monitoring-location/01485755/#parameterCode=62620&period=P7D&showMedian=true.

[3] U.S. Geological Survey. (1997). *Gage height dataset: murderkill River at Bowers, DE-01484085* . USGS current water data for the nation. https://waterdata.usgs.gov/monitoring-location/01484085/#parameterCode=00065&period=P7D&showMedian=false.

[4] Conner W.H., Askew G.R. (1992). Response of baldcypress and loblolly pine seedlings to short-term saltwater flooding. Wetlands.

[5] Maas E.V., Hoffman G.J., Chaba G.D., Poss J.A., Shannon M.C. (1983). Salt sensitivity of corn at various growth stages. Irrigat. Sci..

[6] Solinst Canada Ltd. Levelogger 5 user guide: 1.2.4 conductivity. https://www.solinst.com/products/dataloggers-and-telemetry/3001-levelogger-series/operating-instructions/user-guide/1-introduction/1-2-4-conductivity.php?gad_source=1&gclid=CjwKCAjw-JG5BhBZEiwAt7JR6_mqQCOp9pz9oXY3Sc8tAyIwJwnEoN_InOqVYwWX0YIPk2WwkAhlJhoC9bUQAvD_BwE(2024a).

[7] Standard Methods online – Standard methods for the examination of water and wastewater. http://standardmethods.org/.

[8] Pratt D., McQuiggan R., Bacmeister E.S., Sprague-Getsy A., Michael H. (2025). http://www.hydroshare.org/resource/845670d788eb4385a17b1699bdc0383d.

[9] Solinst Canada Ltd. Levelogger 5 user guide: 8.2 manual barometric compensation. https://www.solinst.com/products/dataloggers-and-telemetry/3001-levelogger-series/operating-instructions/user-guide/8-data-compensation/8-2-manual-barometric-compensation.php?gad_source=1&gclid=Cj0KCQjwwMqvBhCtARIsAIXsZpanRBaaG2p-7nr9bCRPwMhPfSCfgogYF5klf2DEUnv0_6WeSv-kTZ0aAsOOEALw_wcB (2024b).

[10] Andres A.S., He C., McKenna T.E. (2018).

[11] METER Group. TEROS 11/12 User Manual: Calibration (2024). https://publications.metergroup.com/Manuals/20587_TEROS11-12_Manual_Web.pdf.

[12] METER Group. TEROS 11/12 User manual: specifications (2024). https://publications.metergroup.com/Manuals/20587_TEROS11-12_Manual_Web.pdf.

